# Engaging stakeholders to level up COPD care in LMICs: lessons learned from the “Breathe Well” programme in Brazil, China, Georgia, and North Macedonia

**DOI:** 10.1186/s12913-023-10525-4

**Published:** 2024-01-12

**Authors:** Genevie Fernandes, Siân Williams, Peymané Adab, Nicola Gale, Corina de Jong, Jaime Correia de Sousa, KK Cheng, Chunhua Chi, Brendan G. Cooper, Andrew P. Dickens, Alexandra Enocson, Amanda Farley, Kate Jolly, Sue Jowett, Maka Maglakelidze, Tamaz Maghlakelidze, Sonia Martins, Alice Sitch, Aleksandra Stamenova, Katarina Stavrikj, Rafael Stelmach, Alice Turner, Zihan Pan, Hui Pang, Jianxin Zhang, Rachel E. Jordan

**Affiliations:** 1 International Primary Care Respiratory Group, London, UK; 2 Usher Institute, University of Edinburgh, Edinburgh, UK; 3 Institute of Applied Health Research, University of Birmingham, Birmingham, UK; 4 Health Services Management Centre, School of Social Policy, College of Social Sciences, University of Birmingham, Birmingham, UK; 5 Life and Health Sciences Research Institute (ICVS), School of Medicine, University of Minho, Braga, Portugal; 6 PT Government Associate Laboratory, ICVS/3B’s, Braga/Guimarães, Portugal; 7 Department of General Practice, Peking University First Hospital, Beijing, China; 8 Lung Function & Sleep, Queen Elizabeth Hospital, Birmingham, UK; 9 Observational and Pragmatic Research Institute, Midview City, Singapore; 10 Georgian Respiratory Association, Tbilisi, Georgia; 11 Petre Shotadze Tbilisi Medical Academy, Tbilisi, Georgia; 12 Ivane Javakhishvili Tbilisi State University, Tbilisi, Georgia; 13 Family Medicine, ABC Medical School, São Paolo, Brazil; 14 NIHR Birmingham Biomedical Research Centre, University Hospitals Birmingham NHS Foundation Trust and University of Birmingham, Birmingham, UK; 15 Faculty of Medicine, Institute of Social Medicine, Ss. Cyril and Methodius University in Skopje, Skopje, Republic of North Macedonia; 16 Center for Family Medicine, Faculty of Medicine, Ss.Cyril and Methodius University in Skopje, Skopje, Republic of North Macedonia; 17 Pulmonary Division, Heart Institute (InCor), Hospital das Clinicas da Faculdade de Medicina da Universidade de Sao Paulo, São Paolo, Brazil; 18 Department of Emergency, Beijing Friendship Hospital, Capital Medical University, Beijing, China

**Keywords:** Stakeholder engagement, COPD, Research impact, Translation, LMICs

## Abstract

**Background:**

Effective stakeholder engagement in health research is increasingly being recognised and promoted as an important pathway to closing the gap between knowledge production and its use in health systems. However, little is known about its process and impacts, particularly in low-and middle-income countries. This opinion piece draws on the stakeholder engagement experiences from a global health research programme on Chronic Obstructive Pulmonary Disease (COPD) led by clinician researchers in Brazil, China, Georgia and North Macedonia, and presents the process, outcomes and lessons learned.

**Main body:**

Each country team was supported with an overarching engagement protocol and mentored to develop a tailored plan. Patient involvement in research was previously limited in all countries, requiring intensive efforts through personal communication, meetings, advisory groups and social media. Accredited training programmes were effective incentives for participation from healthcare providers; and aligning research findings with competing policy priorities enabled interest and dialogue with decision-makers. The COVID-19 pandemic severely limited possibilities for planned engagement, although remote methods were used where possible. Planned and persistent engagement contributed to shared knowledge and commitment to change, including raised patient and public awareness about COPD, improved skills and practice of healthcare providers, increased interest and support from clinical leaders, and dialogue for integrating COPD services into national policy and practice.

**Conclusion:**

Stakeholder engagement enabled relevant local actors to produce and utilise knowledge for small wins such as improving day-to-day practice and for long-term goals of equitable access to COPD care. For it to be successful and sustained, stakeholder engagement needs to be valued and integrated throughout the research and knowledge generation process, complete with dedicated resources, contextualised and flexible planning, and commitment.

## Introduction

Effective stakeholder engagement is increasingly recognised as critical to ensure research conducted is relevant to local communities and that the emerging knowledge is applied effectively to policy and practice [1]. In health research, stakeholder engagement refers to a process where researchers seek the knowledge and experiences of individuals and groups interested or impacted by a disease, condition or an intervention, and work with them to support, contribute, enable or collaborate in the decision-making processes of research and translation [2]. Stakeholders should be involved in a dialogue throughout the research cycle, from prioritisation through to dissemination and implementation [3], making engagement a process, not an event. Although stakeholder engagement is promoted by health research funding agencies as a pathway for achieving impact [1, 4] it is still a nascent concept, especially for many researchers in low-and middle-income countries (LMICs) [5, 6]. Little has been reported about its process and impacts. Therefore, in this article, we describe the process of stakeholder engagement led by the four country teams in our global “Breathe Well” research programme and discuss the lessons learned.

## Breathe well programme

Chronic Obstructive Pulmonary Disease (COPD) is an incurable and progressive chronic condition that causes disabling breathlessness, cough, increased phlegm production and fatigue [7]. In 2019, COPD accounted for 392 million cases and nearly 3 million deaths worldwide, with more than 75% of these occurring in LMICs [8]. This burden is increasing due to a combination of ageing populations and increasing prevalence of risk factors [9]. Exacerbations, or “flare-ups” of symptoms, are triggered by respiratory infections, pollution and extreme weather, and may result in hospital admissions, increased disability and death. COPD imposes a substantial financial burden on individuals, families and societies through high costs of medical treatment and impact on workplace and home productivity [10–12].

The magnitude and distribution of the population at risk of COPD means that a significant involvement of primary care is the only viable way to adequately manage it, ensuring no one is left behind [13]. However, in many LMICs, diagnosis and management of COPD is mainly limited to secondary and tertiary healthcare facilities, often with high out-of-pocket costs [14]. The Breathe Well programme (Building Research Across the World in Lung Disease) sought to address this gap by collaborating with clinicians from four middle-income countries (Brazil, China, Georgia and the Republic of North Macedonia) to build research capacity to improve COPD prevention, diagnosis and management in primary care settings [15]. Breathe Well was funded by the UK National Institute for Health and Care Research (NIHR) Global Health Research Groups programme, led by the University of Birmingham and facilitated by the International Primary Care Respiratory Group (IPCRG). This research programme (**see** Table 1) was underpinned by specific training, supportive supervision and experiential learning, a key component of which was stakeholder engagement.

**Table 1:** Breathe Well research studies implemented in the four programme countries

Country		Study 1	Study 2
**BRAZIL**	**Aim**	To identify the most cost effective screening strategies for identifying undiagnosed COPD amongst patients (≥ 40 years) with systemic arterial hypertension.	To explore the barriers and enablers to physical activity and exercise programmes amongst COPD patients in Brazil with and without anxiety or depression.
	**Methods**	Cross-sectional study with 1,162 participants in 9 basic health units in the São Bernardo do Campo municipality.	Qualitative study in basic health units in the São Bernardo do Campo municipality; 6 focus groups with patients diagnosed with COPD, with/without signs of anxiety or depression.
**CHINA**	**Aims**	To identify the most cost effective screening strategies for identifying undiagnosed COPD in the general population in China.	To explore the management and understanding of COPD reported by patients in China and design a novel community lung health service.
	**Methods**	Cross-sectional study with 2445 subjects in 8 community health centre sites across four regions of China.	Mixed methods study (questionnaires and focus groups) with patients diagnosed with COPD and general practitioners (GP) from 4 cities in China.
**GEORGIA**	**Aim**	To test the feasibility of a culturally tailored pulmonary rehabilitation programme compared to usual care for patients with symptomatic COPD of MRC ≥ 2.	
	**Methods**	Randomised controlled feasibility trial with qualitative interviews with eligible participants in Tbilisi, Georgia.	
**REPUBLIC OF NORTH MACEDONIA**	**Aim**	To assess if additional assessment and communication of lung age/feedback on exhaled carbon monoxide levels among smokers in primary care increases likelihood of quitting smoking compared to giving very brief smoking cessation advice alone.	
	**Methods**	Randomised controlled trial with 1366 participants from 32 GP practices from different parts of Macedonia.	

## Overarching approach for stakeholder engagement

### Objectives and resources

We supported each country team to identify, prioritise and engage with relevant stakeholders including people affected by COPD, clinicians, and local and national policy makers, who could contribute towards the smooth implementation of the research studies, share knowledge, as well as influence the uptake of positive findings into policy and practice. This approach was designed to be practical, building on existing skills and knowledge, and enabling researchers and stakeholders to contribute in feasible ways with a clear purpose.

### Preliminary activities

Initially, groups of 4–8 patients, clinicians, public health managers and policy-makers were identified by each country team to rank research needs through a formal prioritisation exercise guiding the design of the research studies [16]. Patient members were invited from different gender, age and socio-economic groups, and were prepared to contribute to the research prioritisation exercise in two ways. First, country teams organised preparatory meetings with patient members to discuss the prioritisation exercise, their role in this process, and to clarify any questions. Second, materials for this exercise including 10 research study topics and accompanying evidence vignettes for each, was translated and shared with all the participants including patient members, in advance, which enabled them to participate fully. University of Birmingham team members visited all four countries to promote and oversee the prioritisation exercise. Scoping interviews with each country team explored existing engagement capacities and formal and informal mechanisms for influencing policy and identifying relevant stakeholders.

### Tailored country plans

Based on findings from the preliminary activities and guided by relevant literature [1, 3, 17, 18], an overarching protocol was designed to guide tailored stakeholder engagement plans adapted to each country’s needs and context. Facilitators employed by IPCRG oriented the country teams to the protocol and offered training and support to develop individual country plans over 6–9 video conferencing sessions. Country teams first used the 9 C stakeholder analysis model adapted by IPCRG [19] to identify stakeholders who could offer relevant knowledge or contribute to the Breathe Well studies. Each team prioritised the most relevant stakeholders using the Power and Impact matrix [20, 21]. Next, specific objectives and methods for engaging each relevant stakeholder were outlined, being flexible to adapt to changing socio-political, economic and other structural factors. For example, unlike in high-income countries [22], as there was a lack of respiratory patient support groups in the four Breathe Well countries, systematic engagement with patients was difficult at the beginning and relationships had to be built over time. Elections across countries resulted in a change in political power; those with potential power due to their knowledge or experience often did not recognise it or have an organised “voice” (e.g. primary care physicians and patients); and certain stakeholders (e.g. journalists) were strategically included later in the plan when messages had been crafted and tested for the key audiences.

## Country case studies

### Brazil

Breathe Well researchers in Brazil studied effective COPD screening and management strategies that could be delivered in primary care facilities [23]. There is limited data on patients with COPD in Brazil as this disease is not yet widely diagnosed in primary care, so there was a need to identify alternative strategies for participant recruitment for the studies. As primary health care in the country already has the mandate to manage cardiovascular disease (CVD) and hypertension, and since there is frequent comorbidity between CVD and COPD, the team conducted their study among patients living with hypertension. Researchers identified patients and primary care providers from existing networks and invited them to participate in a project advisory group, which was consulted throughout the studies.

Typically, public health programmes have been reported to work in a siloed approach, often lacking horizontal coordination [24]. Unusually, the Breathe Well team of primary care clinicians already had strong connections with the state-level managers of the government tobacco control programme and used these established networks to identify and approach representatives from the primary care and non-communicable diseases programmes in the São Paulo State Health Department and the Federal Ministry of Health. The team also engaged and sought advice from existing contacts from selected non-governmental agencies and respiratory disease guideline implementation groups such as Global Initiative for Asthma (GINA) Brazil and Global Initiative for Chronic Obstructive Lung Disease (GOLD) Brazil, and approached new stakeholders from the Pan American Health Organization to disseminate findings at the regional level.

A distinctive feature of the Brazilian team’s plan was a whole-of-society approach, bringing together government and non-government stakeholders in workshops rather than engaging stakeholders separately. This approach spurred interest and collaboration from policy stakeholders including from the Ministry of Health in developing a manifesto and a proposal for a national action plan for COPD in Brazil covering surveillance, prevention and health promotion, and comprehensive care.

A patient testimonial video was produced for advocacy, while research findings were used to develop practical training programmes for primary care providers on COPD screening and multidisciplinary management. Importantly, identification and registration of COPD patients has now been integrated into the care pathway protocol in primary health care facilities in the study site of São Bernardo do Campo municipality and plans for implementation across Brazil are underway, supported by the Ministry of Health. Further, the partnership between the primary care team and the public tobacco control unit ensures that individuals newly diagnosed with COPD receive tobacco cessation support as first line treatment and not just prevention [25]. While industry partners were engaged separately from government stakeholders to prevent any conflict of interest, they were supportive of investing in and improving COPD management in the country, and funded stakeholder engagement meetings after the Breathe Well programme ended.

### China

Despite the high burden of people with undiagnosed COPD (estimated 90 million) [26] and the national priority [27] for early identification of this disease, primary care providers still lack the capacity, incentives and tools to diagnose and treat COPD [28, 29]. Breathe Well researchers in China assessed cost-effective screening strategies for identifying undiagnosed COPD in primary care [30] and explored management among patients and general practitioners (GPs).

Patients from the research sites were invited to a patient advisory group and a project steering committee, and were consulted throughout the studies. With inputs from patients, GPs and influential neighbourhood committees (local self-governance units) linked with community health centres in study sites of Xicheng and Haidian districts in Beijing, the team developed an educational package on COPD prevention, symptoms and timely care-seeking in the nearest primary care facilities. Patient groups and neighbourhood committees disseminated these resources through community bulletin boards and WeChat (social media platform).

Participating GPs from the study sites were trained and certified in an expert-led workshop covering study processes and COPD assessment techniques including spirometry lung function tests, strengthening primary care capacity and improving clinical practice. The team kept the regional groups of the Chinese Alliance for Respiratory Diseases in Primary Care informed about research developments and findings through meetings, conferences and widespread social media networks. Participating in Breathe Well facilitated the team in securing funding of 2.15 million yuan (£261,225) from the Department of Primary Health (National Health Commission of the People’s Republic of China), the Alliance and Peking University First Hospital to continue their research on prevention and management of COPD in primary care and the community. As the Alliance reports directly to the national clinical director of respiratory care, the team were able to discuss research implications at government level. Importantly, the team has now adopted the practice of stakeholder engagement for their ongoing research studies.

### Georgia

The Georgian team investigated the effectiveness of a culturally tailored pulmonary rehabilitation (PR) programme [31]. As GPs are not incentivised to diagnose COPD in Georgia, the team found it difficult to identify participants through existing primary care networks and had to subsequently conduct the study through a secondary hospital with existing links with patients with COPD.

The team engaged patients and caregivers through a trial steering committee and the newly set up ‘Lung health club,’ a support group on Facebook with 3100 members, offering information about lung health, quitting tobacco, and care seeking behaviours. The level of trust by the community in the country research team who have a high profile nationally, including television appearances, and the lack of any usual ongoing care for chronic respiratory problems, contributed to the significant community buy-in for the club. The club serves as a virtual community for sharing knowledge and experiences of living with COPD, with regular informational text, images, videos, and practical resources. The principal investigators also regularly featured on television programmes introducing and discussing the benefits of PR.

The team trained healthcare providers in spirometry, developed a practical handbook on PR, and disseminated their findings in lectures at a medical teaching university and within the scientific community and professional medical associations across the country. Professional pulmonology and cardiology associations have since agreed to raise awareness about this service and supported discussions for a potential cardio-pulmonary rehabilitation programme. Being executive members of Georgian Respiratory Association, the team marshalled this group to include a PR module in an accredited training programme for 200 doctors across the country.

Using longstanding networks, the principal investigators were able to set up meetings with federal leaders from the Georgian Parliament, the Ministry of Health, and the National Center for Disease Control and Public Health. The team built on health leaders’ interest in breathlessness treatment and recovery due to SARS-CoV-2 to attract attention to PR as an affordable intervention that could be delivered through the public health programme. Drawing from the study findings, the team has set up a PR programme in their own private healthcare practice financed through user fees. Although, as PR is not covered by national health insurance, cost remains a barrier in the uptake of this service. The team, in their roles as members of the Georgian Respiratory Association, continue to advocate for PR to be delivered through the national public health programme.

### Republic of North Macedonia

Smoking is highly prevalent and culturally acceptable in the Republic of North Macedonia; tobacco farming contributes significantly to the national revenue [32]. Cessation services and pharmacological treatment are not covered by health insurance or adequately available in the country, making quitting difficult. As smoking is the main risk factor for COPD in countries with high tobacco dependence, the Breathe Well team tested a smoking cessation intervention delivered in primary care [33].

Patients were invited to a trial steering committee contributing practical knowledge that supported research implementation; they also gained knowledge about smoking cessation through informational leaflets prepared by the research team. This improved flow of knowledge between patients and researchers was novel given the lack of patient involvement in research in the country. The team used their existing networks of GPs to recruit participants from 32 practices in diferent parts of Macedonia; training them in the study process and assessment techniques. Further, a larger group of GPs were trained in smoking cessation through face to face, practical teaching and an online webinar by the Center for Family Medicine and Faculty of Medicine in collaboration with the Institute of Public Health. To address the lack of observable “buy-in” by health care professionals to the importance of treating tobacco dependence, additional online, awareness and skill building workshops were organised for health professionals as well as community-based nurses who were engaged through the Macedonian Nurses Society.

Given the high rates (29%) of smoking among doctors [34], the team partnered with the Medical Students Association in a leading university and conducted in-person and online training workshops for medical students on tobacco cessation and skill building in delivering such services. They have also applied for accreditation for an elective subject on ‘tobacco control and smoking cessation,’ and are hopeful for this to be included in the undergraduate medical curriculum of the university. At the federal level, the team have used Breathe Well as an entry point to advocate for primary care reforms to strengthen family medicine and reimburse smoking cessation services. At the regional level, the principal investigator was invited by the World Health Organization (WHO) to discuss their findings for sensitising policy makers to the challenges of helping people quit without pharmacotherapy [35].

## Lessons learned and challenges faced

The core competencies of primary care teams such as person-centred care, community orientation, problem solving, comprehensive management and a holistic approach to care [36] facilitates their ability to readily engage and work with stakeholders. Our experience demonstrated that primary care teams can successfully engage with a wide range of stakeholders to effectively implement research. This was possible because stakeholder engagement was a valued component of the programme complete with dedicated human and financial resources; country teams’ researchers were encouraged and mentored to develop tailored stakeholder engagement plans with flexibility to adapt to the changing political, economic and social environments. Importantly, through their persistent engagement, even after the programme ended, the clinician-researchers demonstrated their commitment for translating the knowledge generated from the research studies into practice and striving for equitable access for COPD care.

WHO recommends four techniques - push, user-pull, exchange, and integrated efforts - for effective knowledge translation with decision-makers and for closing the gap between research and policy [37]. The Breathe Well country teams illustrated the effective use of these techniques in their engagement with stakeholders. The Georgian team tailored their research findings from the PR intervention to align with health policy makers interest in SARS-CoV-2, demonstrating ‘push’ efforts. Medical students from the Republic of North Macedonia reached in or accessed research insights on tobacco cessation after their engagement with the Breathe Well team, illustrating ‘user-pull’ efforts. The Brazilian researchers and policy makers worked collaboratively to develop the national action plan for COPD, highlighting ‘exchange’ efforts. Teams have also used these approaches simultaneously, to work with different stakeholder groups, exhibiting ‘integrated’ efforts (see Table 2).

**Table 2:**
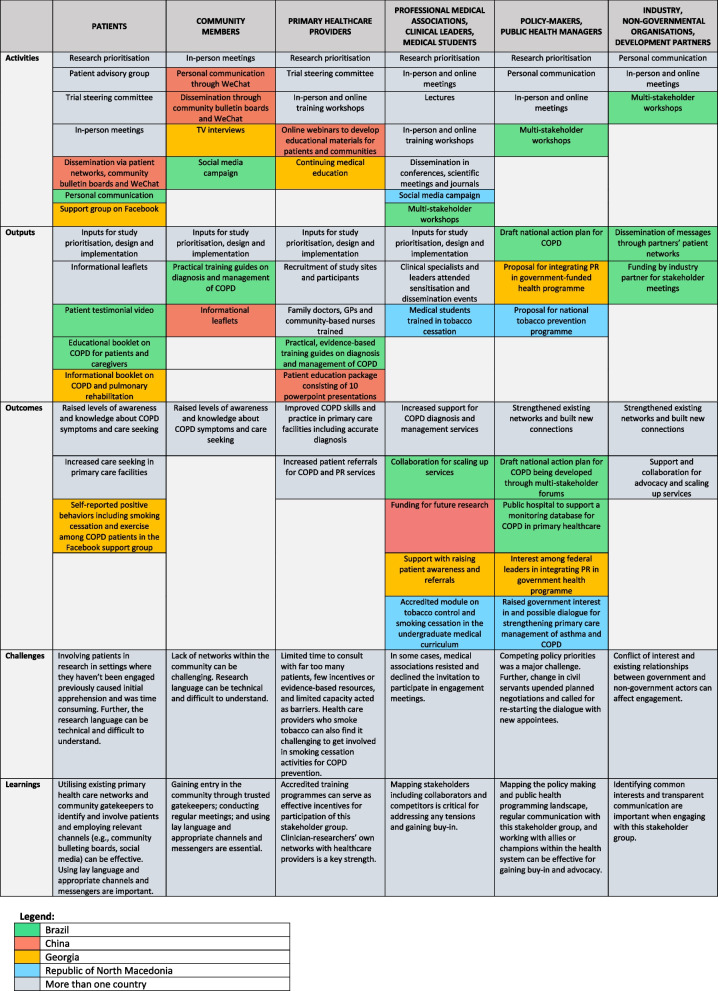
Breathe Well stakeholder engagement process in the four programme countries

Effective stakeholder engagement requires dialogue in a local context and interaction with tacit, experiential knowledge as well as empirical knowledge; not everyone will agree during this process but identifying, acknowledging and addressing tensions can help in tackling barriers and building long-term partnerships. Engagement is a long-term, iterative and arduous process; more stakeholders may emerge during the process and require inclusion; some stakeholders disengage as their roles, power derived from their position or interest change. Despite these inherent challenges, the teams used Breathe Well as an opportunity to cultivate a culture of patient and community involvement, improve healthcare providers’ skills and practice to diagnose and manage COPD, and advocate for resource mobilisation for COPD care.

The lack of patient and public involvement in research and respiratory patient support groups in all four countries was challenging, requiring intensive efforts to identify patients through GP networks and persuade them to join advisory groups and steering committees. Setting up an information and support group on social media was particularly effective and facilitated sustained patient engagement after the study ended. Essentially, local actors’ (patients and clinician researchers) knowledge and expertise were utilised to strengthen patient and public involvement in research, while focusing on the larger goal of equitable access to COPD care.

Healthcare providers were incentivised to participate through continuing medical education programmes and accreditation. Keeping influential actors from government and non-governmental agencies informed throughout the study was critical. The COVID-19 pandemic was a major challenge, severely limiting possibilities for planned in-person engagement with stakeholders. Although, remote technologies were adopted where possible. Change in civil servants within the Ministry of Health also upended engagement plans. Policy makers were focused on pandemic control, but our teams adapted their approach to align with national and international efforts. Finding synergies between COPD and competing policy priorities and appropriately framing the actionable messages emerging from the research was effective in enabling interest and dialogue with decision-makers.

While interest and support for COPD prevention, diagnosis and management in primary care has been generated, inadequate financing currently limits the implementation of these services. GPs and primary care teams need to be financially reimbursed. This will require long-term advocacy and policy reform at the federal level, which can be difficult for researchers to sustain beyond the study funding. Hence researchers will need to build partnerships with allied stakeholders including government, non-government, and private healthcare actors to support the policy dialogue for financing COPD care.

## Conclusion

We illustrate how stakeholder engagement enabled primary care actors to produce and utilise local knowledge relevant for small wins such as improving day-to-day practice as well as for influencing long-term goals of equitable access to COPD care [38]. Primary care clinicians from LMICs transitioned from being stakeholders to research leaders. They used the Breathe Well programme as leverage, reconnected with known stakeholders, built new networks, and initiated new collaborations to sustain the momentum towards improved prevention, diagnosis and care for people with COPD. While achieving meaningful and impactful stakeholder engagement experience is still a learning process, having committed professionals willing and able to build connections and relationships with stakeholders is key for the future of lung health.

## Data Availability

The data generated during the current study can be made available from the corresponding author on request.
